# Perceptions and attitudes toward artificial intelligence among frontline physicians and physicians’ assistants in Kansas: a cross-sectional survey

**DOI:** 10.1093/jamiaopen/ooae100

**Published:** 2024-10-07

**Authors:** Tanner B Dean, Rajeev Seecheran, Robert G Badgett, Rosey Zackula, John Symons

**Affiliations:** Department of Internal Medicine, Intermountain Health, Salt Lake City, UT 84120, United States; Department of Internal Medicine, University of New Mexico Health Sciences Center, Albuquerque, NM 87106, United States; Department of Internal Medicine, University of Kansas School of Medicine—Wichita, Wichita, KS 67214, United States; Center for Clinical Research—Wichita, University of Kansas School of Medicine—Wichita, Wichita, KS 67214, United States; Center for Cyber Social Dynamics, University of Kansas, Lawrence, KS 66045, United States

**Keywords:** artificial intelligence, physicians, trust, surveys and questionnaires, healthcare delivery

## Abstract

**Objective:**

This survey aims to understand frontline healthcare professionals’ perceptions of artificial intelligence (AI) in healthcare and assess how AI familiarity influences these perceptions.

**Materials and Methods:**

We conducted a survey from February to March 2023 of physicians and physician assistants registered with the Kansas State Board of Healing Arts. Participants rated their perceptions toward AI-related domains and constructs on a 5-point Likert scale, with higher scores indicating stronger agreement. Two sub-groups were created for analysis to assess the impact of participants’ familiarity and experience with AI on the survey results.

**Results:**

From 532 respondents, key concerns were Perceived Communication Barriers (median = 4.0, IQR = 2.8-4.8), Unregulated Standards (median = 4.0, IQR = 3.6-4.8), and Liability Issues (median = 4.0, IQR = 3.5-4.8). Lower levels of agreement were noted for Trust in AI Mechanisms (median = 3.0, IQR = 2.2-3.4), Perceived Risks of AI (median = 3.2, IQR = 2.6-4.0), and Privacy Concerns (median = 3.3, IQR = 2.3-4.0). Positive correlations existed between Intention to use AI and Perceived Benefits (*r* = 0.825) and Trust in AI Mechanisms (*r* = 0.777). Perceived risk negatively correlated with Intention to Use AI (*r* = −0.718). There was no difference in perceptions between AI experienced and AI naïve subgroups.

**Discussion:**

The findings suggest that perceptions of benefits, trust, risks, communication barriers, regulation, and liability issues influence healthcare professionals’ intention to use AI, regardless of their AI familiarity.

**Conclusion:**

The study highlights key factors affecting AI adoption in healthcare from the frontline healthcare professionals’ perspective. These insights can guide strategies for successful AI implementation in healthcare.

## Background and significance

Artificial intelligence (AI) technologies, particularly machine learning (ML), are gaining widespread prominence in healthcare due to their potential to enhance various aspects of medical practice.^1–4^ A PubMed search of “artificial intelligence” reveals an exponential increase in AI articles from 4400 articles in 2010 to over 3800 articles in 2022. As the clinical benefits or harms of AI are discovered, understanding physicians’ perceptions and attitudes toward its use in healthcare becomes increasingly crucial for implementation and acceptance. Previous studies have explored the factors influencing physicians’ intention to adopt AI in healthcare.^5–11^ While existing studies have shed light on the attitudes toward AI in healthcare, a gap remains, particularly in quantifying the direct views of frontline healthcare professionals.

The broader AI ethics literature identifies concerns regarding the epistemic opacity of AI, its potential biases arising from biased data, and information privacy as potential hurdles to AI in healthcare.^12^ Concerns about the scientific reliability of AI have also figured in the philosophical literature.^13–15^ It has been shown that implementing innovative technologies into healthcare depends on individuals and their opinions.^16^ To ensure the responsible and ethical implementation of AI in healthcare in our state’s clinical practices, we must begin by quantifying physicians’ concerns and addressing their perspectives as frontline users of this technology.

## Objective

This study aims to gain insight into frontline healthcare professionals’ different attitudes and perceptions regarding AI in healthcare through a statewide survey. The study also examines how these concerns relate to an intention to use AI. Additionally, the impact of participants’ familiarity and experience with AI will be challenged to understand the relationship between perceptions and expertise further. The findings of this study could play a role in shaping future AI implementation strategies and policymaking, ensuring the real concerns and needs of the frontline users are addressed. Our study highlights the nuanced perceptions of physicians toward AI and sheds light on their specific concerns and priorities with respect to these technologies.

## Materials and methods

### Study design

This study is a cross-sectional survey of physicians and physicians’ assistants in the state of Kansas.

The relevant institutional ethics committee approved the study protocol and procedures: KUMC (University of Kansas Medical Center) Institutional Review Board # STUDY00149585. We ensured informed consent, confidentiality, and data protection throughout the study. For reporting the results of our study, we followed the Consensus-Based Checklist for Reporting of Survey Studies (CROSS) checklist (see Supplementary material), designed for reporting survey studies and can be found on the EQUATOR Network website.^17^

### Participant recruitment

To obtain a comprehensive sample of participants, an email invitation was sent to all 12 290 actively licensed physicians and physician assistants of The Kansas State Board of Healing Arts (KBHA). The KBHA is the state of Kansas’ medical licensing and regulatory board for 16 different health professions. Email addresses were obtained through The KBHA contact database includes licensed physicians (M.D. and D.O.), physician assistants, residents, and fellows who opt into having their contact information made available upon request. The survey was conducted from February 2023 to March 2023 with 2 email reminders during this period. A sample size of 373 was determined for a population of 12 290, 5% margin of error, and 95% confidence level.

### Data collection

Study data were collected and managed using REDCap electronic data capture tools hosted at the University of Kansas Medical Center.^18^^,^^19^ All redcap responses were entered anonymously. For data to be entered into the survey database it must be associated with an invitation sent via email. The survey was designed to allow only one response per invitation link to prevent multiple responses. The survey data were maintained on a local encrypted research drive hosted by the University of Kansas Medical Center. Access to the raw data was maintained through REDCap and limited to those participating in the statistical analysis of the study.

### Survey instrument

We utilized an existing, validated, survey by Esmaeilzadeh with minor changes to the demographics section.^12^ We used the survey under a Creative Commons Attribution 4.0 International License. This survey, tailored for AI in healthcare, was chosen for its previously validated constructs that encompass a broad spectrum of potential concerns relevant to front-line physicians. The constructs within the survey had also been shown to be major influencing ideas for intention to use AI.^12^ This survey was also relevant to our population of respondents with varying experiences with AI. It showed, through structured equation models, direct influence between respondents’ familiarity and technical knowledge about AI and their perceived intention to use AI.^12^

The survey instrument consisted of 3 domains (Technological concerns, Ethical concerns, and Regulatory concerns). Each domain was formed by 2-3 constructs. Constructs with a negative perception were performance anxiety, social biases, privacy concerns, communication barriers, unregulated standards, and liability issues associated with AI in healthcare. Positive constructs were trust in AI mechanisms, intention to use AI, and benefits of AI. Each construct consisted of several individual questions referred to as items. There were 54 items across all constructs. Apart from the survey items, demographic information, self-reported computer skills, self-rated technical knowledge about AI, and prior experience with AI were also reported.

### Comparison of AI naive and experienced participants

Two sub-groups were created for analysis to assess the impact of participants’ familiarity and experience with AI on the survey results. The AI naïve subgroup was defined as those who rated themselves as having the lowest or no computer skills, lowest or no technical knowledge about AI, having no previous experience with AI-enabled services or devices, no familiarity with AI-based devices/programs used for clinical purposes, and no familiarity with AI-enabled health services. The responses of AI naive participants were then compared against all other respondents to identify any significant differences in domain and subdomain perceptions.

To compare participants’ level of experience with AI, a composite score was constructed from self-selected responses to a series of survey items. AI naïve was assigned if each response to the following survey questions were true (else AI experience was assigned):

Responding “No” to:

- *Have you ever used any AI-enabled services or devices for any reason except for healthcare?*
- *Have you ever used any AI-enabled health services? (Such as AI embedded in smart medical devices)*

Responding “Not at all” to:

- *Generally, how familiar are you with an AI-based device (used for any purposes except for healthcare)?*

Responding “Terrible” or “Poor” to:

- *How do you rate your technical knowledge about AI?*

This composite accounts for respondents’ experience, familiarity, and technical knowledge about AI. This is similar to the results of structured equation modeling from the original survey showing that technical knowledge about AI and familiarity with AI was significantly associated with the outcome of intention to use AI.^12^

### Data analysis

Participants who submitted responses to the survey were deemed “completers” while those who exited the survey before submitting their responses were “non-completers.” However, all responses were captured in REDCap and included in analyses. To assess the potential for bias among responders, completers vs non-completers were compared. All responses were summarized per item and reported as frequencies and percentages. Because data were not missing at random, no imputations were conducted.

To be comparable to previous research, item responses were pooled by constructs and summarized using means and standard deviations, along with medians and interquartile ranges.^12^ Further, associations were evaluated with Pearson correlations. Box plots were conducted showing the median value of each construct to visually inspect these relationships. Mann-Whitney *U* tests were calculated to compare constructs within subgroups of AI Naïve vs AI Experienced. All statistical analyses were conducted with 2-tailed tests using IBM SPSS Statistics, version 29.

### Ethical consideration

Generative AI (ChatGPT version 3.5 and 4) was used to improve the language and readability of the manuscript and did not replace other research tasks associated with this study. All AI output was critically reviewed and edited by the authors. No data used for this study were created nor modified through generative AI.

## Results

### Participants

Of the 12 290 active members of The Kansas State Board of Healing Arts with available email addresses, 532 responses were received, for a response rate of 4.3%. A total of 519 respondents consented to participate in the study.

Participant responses for those who consented were compared, including 394 (75.9%) completers and 125 (24.1%) non-completers (Supplementary material 1). No observable differences between groups were found. However, of the non-completers, 50 consented but never responded to any question; 75 only responded to the first 2 questions. Subsequently, participation in the survey declined.

Demographic information provided by responders who completed the survey (*n* = 394) was summarized to provide context for the respondent’s backgrounds and expertise in AI (Table 1). Participants were comprised of trainees (5.3%), physicians (80.8%), physicians’ assistants (13.2%) and retired (<1%). The majority of respondents, 44.9% (172 of 383), were aged 46-65 years, 56.2% were male, and 60.3% were White. With regard to lack of AI expertise, 24.9% reported they were not at all familiar with AI-based devices outside of healthcare, 42.0% reported not at all familiar with devices or programs used for clinical purposes, and 32.2% rated their technical knowledge about AI as terrible or poor.

**Table 1.** Demographic information of responders with complete surveys.

			Completed survey responses	
Survey item		*N*	*n* = 394	%
What is your age?		383		
	23-35		60	15.7
	36-45		104	27.2
	46-65		172	44.9
	>65		47	12.3
With which gender do you identify?		381		
	Male		214	56.2
	Female		154	40.4
	Prefer not to answer		13	3.4
What is your race? (choose all that apply)		383		
	White/Caucasian		313	60.3
	Black/African-American		11	2.1
	Native American or American Indian		2	0.4
	Asian/Pacific Islander		31	6.0
	Other (none of the above)		26	5.0
What is your ethnicity?		382		
	Non-Hispanic		352	91.9
	Hispanic		10	2.6
	Declined		21	5.5
What is your current occupation?		380		
	Resident		14	3.7
	Fellow		6	1.6
	Physician		307	80.8
	Physician assistant		50	13.2
	Retired		3	0.8
What is your current practice focus (choose one)		326		
	Anesthesiology		18	5.5
	Family Medicine		79	24.2
	Internal Medicine		41	12.6
	Internal Medicine/Pediatrics		7	2.1
	Obstetrics and Gynecology		11	3.4
	Orthopedic Surgery		12	3.7
	Pediatrics		29	8.9
	General Surgery		11	3.4
	Diagnostic Radiology		9	2.8
	Cardiology		8	2.5
	Pulmonology		5	1.5
	Critical Care/Neurocritical Care/Cardiac Critical Care		10	3.1
	Advanced Surgical Fellowship		6	1.8
	Hematology and Oncology		5	1.5
	Emergency Medicine		13	4.0
	Otolaryngology		6	1.8
	Pathology		6	1.8
	Psychiatry		15	4.6
	Other^a^		35	10.7
If a practicing physician, how many years of experience do you currently have?		307		
	<5 years		34	11.1
	5-10 years		50	16.3
	10-20 years		68	22.1
	>20 years		153	49.8
	Not currently practicing		2	0.7
What is your current work environment (choose all that apply)?		392		
	Metropolitan		221	56.1
	Rural		69	17.5
	Combined		85	21.6
	Not applicable		17	4.3
What best describes your current practice type?		381		
	Outpatient		127	33.3
	Mostly outpatient with some inpatient		80	21.0
	Split outpatient and inpatient		73	19.2
	Mostly inpatient with some outpatient		41	10.8
	Inpatient		55	14.4
	Not currently practicing		5	1.3
What best describes your current practice environment?		379		
	Private practice		106	28.0
	Not-for-profit system		207	54.6
	For-profit system		66	17.4
Yes, I practice in an academic setting (overseeing learners)			197	51.8
Yes, I have used other AI-enabled services or devices (for any reason except for healthcare)		378	151	39.9
Generally, how familiar are you with an AI-based device (used for any purposes except for healthcare)?		381		
	Not at all familiar		95	24.9
	Slightly experienced		185	48.6
	Moderately experienced		80	21.0
	Very experienced		14	3.7
	Extremely experienced		7	1.8
I have used any AI-enabled health services? (Such as AI embedded in smart medical devices)		379	110	29.0
How familiar are you with these AI-based devices or programs used for clinical purposes?		381		
	Not at all familiar		160	42.0
	Slightly experienced		151	39.6
	Moderately experienced		53	13.9
	Very experienced		13	3.4
	Extremely experienced		4	1.0
Overall, do you think your health information is		378		
	Sensitive		291	77.0
	Non-sensitive		61	16.1
	No idea		26	6.9
How do you generally rate your computer skills		380		
	Terrible		2	0.5
	Poor		5	1.3
	Average		105	27.6
	Good		164	43.2
	Excellent		104	27.4
How do you rate your technical knowledge about AI?		379		
	Terrible		27	7.1
	Poor		95	25.1
	Average		162	42.7
	Good		77	20.3
	Excellent		18	4.7
Yes, I would you like to participate in a future focus group to discuss AI in medicine?		381	140	36.7

a Gastroenterology, Sports Medicine, Advanced Radiology, Infectious Disease, Endocrinology, Dermatology, Neurological Surgery, Neurology, Physical Medicine and Rehabilitation, Plastic Surgery, Radiation Oncology, Urology, Vascular Surgery, and “Other” with less than 5 respondents.

Abbreviation: AI, artificial intelligence.

### Item responses

Among individual survey items for completers (see Supplementary material 1), the ones for which more than one-third of respondents selected “strongly agree” were: I am concerned that AI devices may decrease human aspects of relations in the medical contexts (36.8%), I am concerned because it is not clear who is responsible when errors result from the use of AI clinical tools (36.0%), I am concerned because it is not clear who becomes responsible if AI-based tools offer wrong recommendations (36.0%), I am concerned that by using AI devices, I may lose face-to-face cues and personal interactions with physicians (35.8%), I am concerned about the lack of clear guidelines to monitor the performance of AI tools in the medical context (34.3%), I am concerned about the liability of using AI-based services for my healthcare (34.3%), I am concerned that the safety and efficacy of AI medical tools are not regulated clearly (33.2%), I am concerned that special policies and guidelines for AI tools are not transparent yet (33.0%), and I am concerned because it is unclear where the lines of responsibility begin or end when AI devices guide clinical care (33.0%).

Interestingly, there were no survey items where more than one-third of respondents selected “strongly disagree.” The survey item with the highest percentage of respondents selecting “strongly disagree” was *I trust that AI-based tools can adapt to specific and unforeseen medical situations* (21.1%), with all other items having < 15% of respondents selecting “strongly disagree.”

The survey items with the highest percentage of selection by all respondents were, *I believe AI-based services can improve diagnostics* (Agree, 55.6%), *using AI-based tools for healthcare purposes is something I would consider* (Agree, 50.8%), and *I am concerned that the medical decisions made by AI devices may be inadequate* (Agree, 49.2%).

### Assessment of constructs

**Figure 1.**
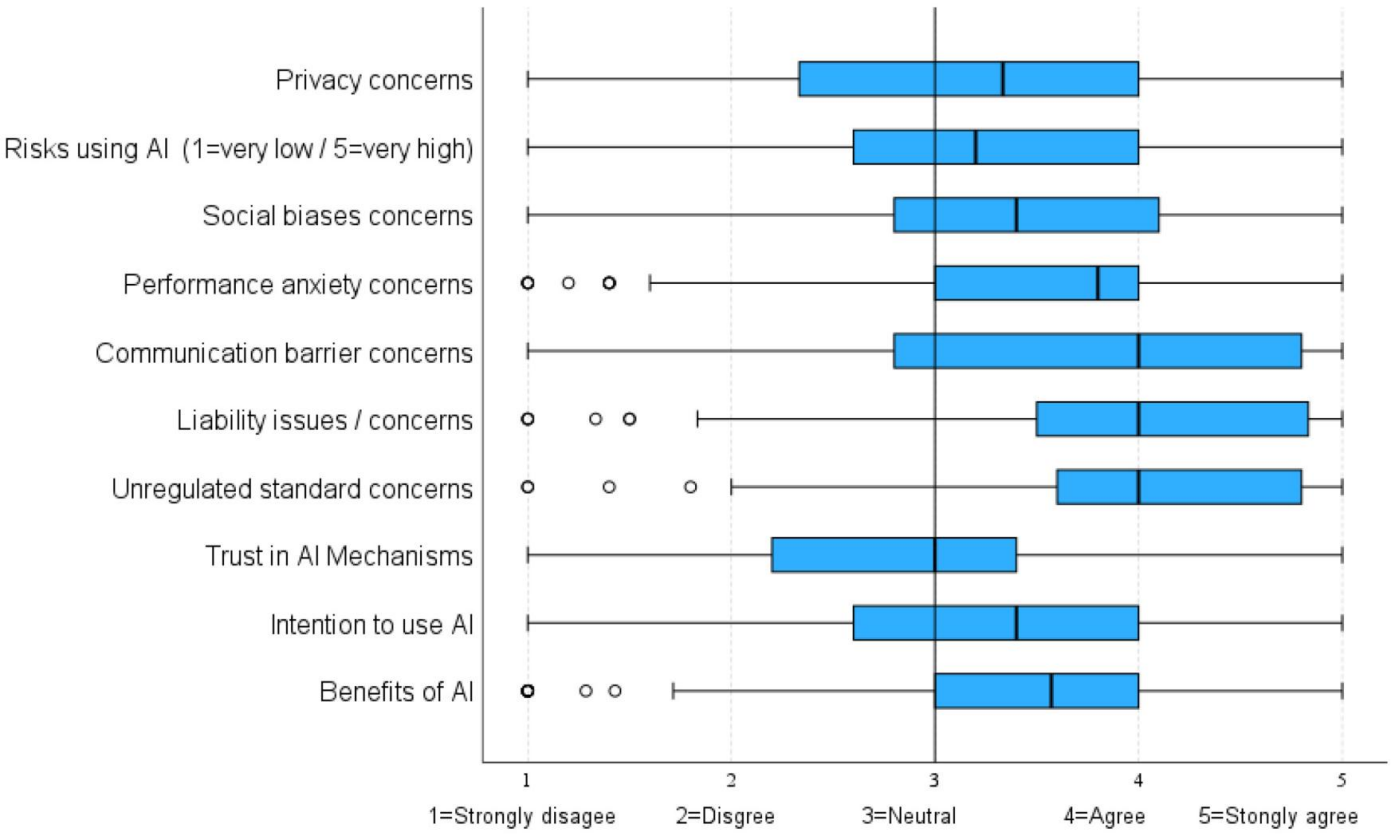
Comparison of responses by constructs. Each box represents the interquartile range, with the outmost edges representing the first quartile (Q1) and third quartile (Q3).

The mean, median, standard deviation, and interquartile ranges of constructs were calculated and compared to identify variations in perceptions (Figure 1). Respondents tended to demonstrate more concern over 3 negative constructs: Perceived Communication Barriers (median = 4.0, IQR = 2.8-4.8), Perceived Liability Issues (median = 4.0, IQR = 3.5-4.8), and Perceived Unregulated Standards (median = 4.0, IQR = 3.6-4.8). Conversely, the more positive perceptions of AI included Perceived Trust in AI Mechanisms, which tended to be rated as neutral (median = 3.0, IQR = 2.2-3.4); Intention to Use AI-based Tools, slightly more favorable (median = 3.4, IQR = 2.6-4.0); and Perceived Benefits (median = 3.6, IQR = 3.0-4.0), which ranged from neutral to agreement.

### Associations among constructs

Correlation analyses were conducted to evaluate associations among the constructs (Table 2). All correlations reported in Table 2 were significant at *P* < .001. The strongest positive correlation observed was between the Intention to Use AI and Perceived Benefits (*r* = 0.825), indicating that respondents tended to agree with both. Another strong relationship was observed between Anxiety and Risks (*r* = 0.726), demonstrating that respondents tended to agree with perceived performance anxiety of AI (inaccurate predictions, medical errors, malfunctions, inadequate medical decisions) and perceived risks (adverse consequences, high likelihood of unexpected problems, degree of uncertainty). The strongest negative correlation was between Perceived Risks of AI and Trust in AI mechanisms (*r* = −0.756), demonstrating that those who believe AI poses risk are less likely to trust in AI mechanisms (for healthcare delivery, diagnostics, ability to adapt to specific and unforeseen medical situation). Another strong negative association occurred between Perceived Risks of AI and Intention to use (*r* = −0.718), such that where respondents rated risk high, they were less likely to use AI.

**Table 2.** Correlation among constructs.

	Anxiety (PRA)	Biases (PSB)	Privacy (PPC)	Trust (PMT)	Communication barrier* (PCB)*	Unregulated (PUS)	Liability (PL)	Risks (PR)	Benefits (PB)
Biases (PSB)	0.595	—							
Privacy (PPC)	0.506	0.417	—						
Trust (PMT)	(−0.656)	(−0.494)	(−0.463)	—					
Communication barrier* (PCB)*	*0.569*	*0.458*	*0.470*	*(−0.528)*	—				
Unregulated (PUS)	0.597	0.545	0.544	(−0.515)	*0.525*	—			
Liability (PL)	0.587	0.490	0.529	(−0.566)	*0.634*	0.688	—		
Risks (PR)	0.726	0.570	0.535	(−0.756)	*0.622*	0.587	0.639	—	
Benefits (PB)	(−0.591)	(−0.442)	(−0.426)	0.679	*(−0.539)*	(−0.403)	(−0.480)	(−0.620)	—
Intention to use (INT)	(−0.634)	(−0.479)	(−0.459)	0.777	*(−0.607)*	(−0.477)	(−0.562)	(−0.718)	0.825

All correlations reported in this table are significant at *P* < .001.

Positive numbers would indicate similar responses, and negative numbers indicate that as 1 construct mean goes up, the other goes down.

For example, PCB vs PB = −0.539, indicating that as ratings increase for PCB, they tend to decline for PB.

Thus, if responding strongly agree (5) to *I am concerned that AI tools may eliminate the contact between healthcare professionals and patients*, respondent may have also responded strongly disagree (1) to *I believe AI-based services can improve diagnostics*.

Abbreviations: AI, artificial intelligence; PSB, perceived social biases; PPC, perceived privacy concerns; PMT, perceived mistrust in AI mechanisms; PCB, perceived communication barriers; PUS, perceived unregulated standards; PL, perceived liability issues; PR, perceived risks; PB, perceived benefits.

### AI naïve vs AI experienced subgroups

Demographics between the 2 subgroups were compared and are shown in Supplementary material 2; the AI naïve group had a higher proportion of women (59.7%) as compared to the AI experienced group (60.5% male). Table 3 summarizes the constructs with comparisons by level of experience with AI; no statistically significant differences were observed.

**Table 3.** Construct comparisons by AI naïve and AI experienced subgroups.

	Naïve		Experienced		
Construct	Median	IQR	Median	IQR	*P*
Intention to use	3.20	(2.8, 3.5)	3.40	(2.6, 4.0)	.204
Perceived benefits	3.57	(3.4, 3.7)	3.62	(3.0, 4.0)	.383
Communication barriers	4.00	(3.1, 4.8)	4.00	(2.8, 4.8)	.207
Liability concerns	4.00	(3.8, 4.6)	4.00	(3.4, 4.8)	.953
Trust in AI mechanisms	3.00	(2.4, 3.1)	3.00	(2.0, 3.5)	.901
Privacy concerns	3.00	(2.3, 4.0)	3.50	(2.3, 4.2)	.167
Risks associated with AI	3.20	(3.0, 3.8)	3.20	(2.6, 4.0)	.734
AI performance anxiety	3.80	(3.4, 4.0)	4.00	(3.0, 4.0)	.988
Social biases	3.50	(3.0, 4.0)	3.40	(2.6, 4.2)	.496
Unregulated standards	4.00	(3.8, 4.4)	4.00	(3.6, 5.0)	.642

Abbreviation: AI, artificial intelligence.

## Discussion

The findings reveal that frontline healthcare practitioners have distinct perceptions about AI in healthcare. The 3 most selected items from the survey, I believe AI-based services can improve diagnostics (Agree, 55.6% of all respondents), using AI-based tools for healthcare purposes is something I would consider (Agree, 50.8% of all respondents), and I am concerned that the medical decisions made by AI devices may be inadequate (Agree, 49.2% of all respondents), present an interesting conundrum. While physicians can see the potential benefits of AI and may even be optimistic about its use in healthcare, they also think that it could fall short in other aspects of medical decision-making. This is congruent with the perception that current AI technologies might excel in identifying patterns used for diagnosis (imaging scans, large datasets, or highly defined diagnostic criteria) but may not excel in the dynamic environments of patient-physician interaction that require multi-factorial decision-making. When making medical decisions, the ability to understand the patient’s medical history, present symptoms, psychosocial context, etc, may be assumed too complex for AI by the respondents.

The study uncovered several apprehensions about the use of AI in healthcare. Most notably, the respondents perceived AI as a potential barrier to the human aspect of patient-physician interactions. A sentiment held by a significant portion of respondents, as evidenced by more than one-third expressing strong agreement to the items: I am concerned that AI devices may decrease human-aspects of relations in the medical contexts (36.8%) and I am concerned that by using AI devices, I may lose face-to-face cues and personal interactions with physicians (35.8%). The perceived barrier to communication could be due to a current perceived barrier experienced with electronic health records (EHRs).^20^^,^^21^ EHRs, while beneficial, have been reported to increase physicians’ “screen time” and reduce face-to-face patient interaction.^22^ The everyday perceived barriers associated with EHR use could explain some initial perceptions that AI would hinder patient communication.

The concerns over perceived liability, regulation of AI, and overall perceived risks associated with AI in healthcare emerged as significant themes in our survey. Seven of the 9 survey items, with more than 33% of respondents selecting strongly agree, reflect apprehensions such as the lack of clarity about responsibility when errors result from AI clinical tools, concerns about the liability of using AI-based services, and uncertainty about the regulation of the safety and efficacy of AI medical tools. These perceptions could be traced to the phenomenon known as “Defensive Medicine.” In defensive medicine, physicians make decisions primarily to safeguard against legal liability rather than focusing solely on optimal patient care.^23^^,^^24^ This mindset might be particularly salient with emerging technologies like AI, where the harms associated with potential errors and the regulatory landscape are not yet clearly defined.^25^ Under a defensive medicine framework, physicians might be discouraged from adopting AI and other emerging technologies unless explicit protections and regulations are in place to mitigate liability. Failing to address these concerns could slow the adoption of AI in healthcare, potentially limiting its future benefits.

The data reveal intriguing insights into the perspectives of healthcare practitioners regarding their Intention to use AI. There is a positive correlation between the Intention to Use AI and the Perceived Benefits of AI (*r* = 0.825), indicating that recognizing AI’s potential advantages plays a crucial role in its acceptance. Trust in AI mechanisms is also a significant factor, showing strong positive correlations with Intention to Use AI (*r* = 0.777) and Perceived Benefits (*r* = 0.679). These positive associations suggest that cultivating Trust in AI mechanisms can foster an inclination to utilize AI. Conversely, Trust in AI exhibits negative associations with Perceived Risks (*r* = −0.756), Perceived Liability (*r* = −0.566), Perceived Communication Barriers (*r* = −0.528), and the Perceived Unregulated Standards associated with AI in healthcare (*r* = −0.515). Based on this information, we postulate that Trust in AI is connected to concerns regarding risks, liability, communication hurdles, and lack of regulatory standards. Therefore, strategies to highlight AI’s benefits and address the concerns around risk, liability, communication, and regulation could influence both Trust in AI mechanisms and Intention to Use AI. Specific interventions to address these concerns present themselves as a target for future research.

The perceptions of respondents in our study align with previously reported findings, particularly in areas such as concerns over AI-associated liability^8^ and the belief that AI can enhance diagnosis. Our respondents, like those in prior studies, acknowledged that AI might lead to a reduction in empathic communication^8^ and identified a lack of trust as a central deterrent to AI’s use in medicine.^7^ However, unlike findings from previous research, our participants did not voice concerns about data privacy, nor did they share the sentiment that AI could allow for increased patient interaction time.^7^^,^^8^

Another aspect to consider in this study is the potential influence of respondents’ knowledge and awareness of AI on their perceptions of AI in healthcare. If users lack understanding or are ignorant of the general safety concerns surrounding AI, it could potentially confound the pooled perceptions observed. Interestingly, our analysis revealed no statistically significant difference between the AI naive subgroup and all other respondents regarding their perceptions of AI. This finding suggests that even individuals with limited computer skills and potentially less awareness of the limitations of AI exhibit similar perceptions toward AI as their counterparts. Further investigation is warranted to validate this observation and to explore the impact of knowledge and awareness on perceptions toward AI in healthcare settings.

This study underscores the nuanced perceptions of healthcare physicians toward AI within the healthcare context, illuminating specific concerns and priorities to address when designing and implementing AI in the medical field. While the Intention to use AI-based tools is significantly influenced by perceived benefits and trust in the technology’s mechanisms, practical concerns related to liability, risk mitigation, regulation of AI, and patient-physician communication also play a critical role. It is imperative to address these practical components alongside enhancing AI’s perceived benefits. By aligning AI development with the aspects that frontline practitioners find most relevant and addressing these diverse needs, greater acceptance and utilization of AI technologies can be fostered in healthcare settings.

The state of Kansas offers an insightful context for understanding physicians’ perceptions toward AI in healthcare. The state boasts a blend of urban and rural environments, each presenting distinctive healthcare demands and obstacles.^26^ This diversity potentially allows the findings to generalize with similar environments nationally, and perhaps internationally. Complementing this, Kansas encompasses both large hospital systems and smaller independent practices, enabling insights to reflect a wide range of healthcare setups. Furthermore, Kansas confronts notable healthcare challenges, such as a shortage of medical professionals, especially in its rural areas, a predicament that mirrors many other locations, supporting the generalizability of our results.

However, it is essential to recognize certain limitations when extrapolating these findings. For instance, the demographic composition of Kansas, although varied, may not perfectly represent other areas, especially those with greater ethnic diversity or differing age profiles. For example, the survey was composed of only 2.1% Black/African-American physicians and only 2.6% ethnically Hispanic frontline physicians. Both numbers are lower than National and Kansas physician data reported by the Association of American Medical Colleges.^27^^,^^28^

### Limitations

A limitation of this research is that the study occurred across a single state in the United States as described prior. Attrition in survey responses was another limitation of this study. There seemed to be a cutoff point where respondents became fatigued with the survey and exited the program. A potential way to decrease this bias in the future is to randomize the presentation of questions to survey participants or validate shorter surveys that maintain the same domains and constructs. Another limitation was the absence of participant self-assessment regarding their experience level, Naïve vs Experienced. While we used a series of questions to determine these categories, we did not inquire whether participants considered themselves naïve or experienced. This aspect might have influenced the composition of the Naïve vs experienced groups and, in turn, affected the conclusion of similar perceptions between these 2 groups. The survey population was skewed toward primary care practitioners (∼30%) which may affect the generalizability to other specialties. The survey’s response rate was 4.3%, which is in the lower range of response rates from other perceptions toward AI in medicine surveys (1.3%-20.4%),^5^^,^^29^^,^^30^ though there are no comparable single state cross-sectional surveys to compare to. The lower response rate may limit the generalizability of the survey results.

## Conclusion

The clinical impact of AI in medicine is a topic of ongoing investigation, with benefits and harms yet to be conclusively defined. This study provides insight into the complex perceptions frontline healthcare practitioners have toward integrating AI within the healthcare setting. The intention to use AI-based tools is influenced by the perceived benefits of the technology and the user’s trust in its mechanisms. Simultaneously, several practical concerns related to regulation, liability, and patient-physician communication were identified. While the clinical benefits and potential harms of AI in medicine continue to be explored, understanding healthcare practitioners’ nuanced perceptions is crucial for developing strategies that maximize the benefits and mitigate the risks of integrating AI into healthcare practice.

Future research is needed to gain deeper insights into physicians’ perceptions of AI and identify practical ways to address these concerns. In addition, work is also required to assess the effectiveness of shorter survey scales to prevent the attrition that was observed in this survey. Finally, further research is needed to explore potential geographic and demographic differences in perceptions toward AI in healthcare to measure the generalizability of these results. These additional research endeavors will add more understanding of the factors influencing physicians’ attitudes and intentions regarding AI adoption in healthcare.

## Supplementary Material

ooae100_Supplementary_Data

## Data Availability

The data underlying this article will be shared on reasonable request to the corresponding author.
