# Supplementary material for: Perceptions and attitudes toward artificial intelligence among frontline physicians and physicians’ assistants in Kansas: a cross-sectional survey
Source: JAMIA Open. 2024 Oct 7;7(4):ooae100. doi: 10.1093/jamiaopen/ooae100 (PMC11458514; doi:10.1093/jamiaopen/ooae100)
Supplement: ooae100_Supplementary_Data [file ooae100_supplementary_data.zip › Supplement 2.docx]

**Appendix 2**. Description of respondent characteristics by AI Naïve status.

|  |  | AI naive | | AI experienced | |
| --- | --- | --- | --- | --- | --- |
| Characteristics | | **n=68** | **%** | **456** | **%** |
| What is your age? | |  |  |  |  |
|  | 23 to 35 | 11 | 16.4 | 49 | 15.5 |
|  | 36 to 45 | 18 | 26.9 | 87 | 27.4 |
|  | 46 to 65 | 26 | 38.8 | 146 | 46.1 |
|  | > 65 | 12 | 17.9 | 35 | 11.0 |
| With which gender do you identify? | |  |  |  |  |
|  | Male | 24 | **35.8** | 190 | **60.5** |
|  | Female | 40 | **59.7** | 114 | **36.3** |
|  | Prefer not to answer | 3 | 4.5 | 10 | 3.2 |
| White / Caucasian | | 57 | 83.8 | 256 | 56.8 |
| Black / African-American | | 2 | 2.9 | 9 | 2.0 |
| Native American or American Indian | | 0 | 0.0 | 2 | 0.4 |
| Asian / Pacific Islander | | 3 | 4.4 | 28 | 6.2 |
| ethnicity | |  |  |  |  |
|  | Non-Hispanic | 65 | 95.6 | 287 | 91.1 |
|  | Hispanic | 1 | 1.5 | 9 | 2.9 |
|  | Declined | 2 | 2.9 | 19 | 6.0 |
| What is your marital status? | |  |  |  |  |
|  | Single | 9 | 13.2 | 28 | 8.9 |
|  | Married | 54 | 79.4 | 259 | 82.2 |
|  | In a relationship | 3 | 4.4 | 14 | 4.4 |
|  | Prefer not to disclose | 2 | 2.9 | 14 | 4.4 |
| What is your immigration background? | |  |  |  |  |
|  | U.S. citizen | 65 | 95.6 | 296 | 94.9 |
|  | Immigrant Visa holder | 0 | 0.0 | 1 | 0.3 |
|  | Non-immigrant Visa holder | 1 | 1.5 | 3 | 1.0 |
|  | Green Card holder | 0 | 0.0 | 2 | 0.6 |
|  | Prefer not to answer | 2 | 2.9 | 10 | 3.2 |
| What is your current occupation? | |  |  |  |  |
|  | Resident | 2 | 2.9 | 12 | 3.8 |
|  | Fellow | 1 | 1.5 | 5 | 1.6 |
|  | Physician | 52 | 76.5 | 255 | 81.7 |
|  | Physician Assistant | 11 | 16.2 | 39 | 12.5 |
|  | Retired | 2 | 2.9 | 1 | 0.3 |
| If a practicing physician, how many years of experience do you currently have? | | | |  |  |
|  | less than 5 years | 8 | 14.8 | 26 | 10.3 |
|  | 5 to 10 years | 8 | 14.8 | 42 | 16.6 |
|  | 10 to 20 years | 8 | 14.8 | 60 | 23.7 |
|  | > 20 years | 30 | 55.6 | 123 | 48.6 |
|  | Not currently practicing | 0 | 0.0 | 2 | 0.8 |
| Current work environment (choose all that apply) | |  |  |  |  |
|  | Metropolitan | 41 | 65.1 | 180 | 39.5 |
|  | Rural | 14 | 22.2 | 55 | 12.1 |
|  | Combined | 11 | 17.5 | 74 | 16.2 |
|  | Not applicable | 5 | 7.9 | 12 | 2.6 |
| What best describes your current practice type? | |  |  |  |  |
|  | Outpatient | 20 | 29.4 | 107 | 34.2 |
|  | Mostly outpatient with some inpatient | 12 | 17.6 | 68 | 21.7 |
|  | Split outpatient and inpatient | 15 | 22.1 | 58 | 18.5 |
|  | Mostly inpatient with some outpatient | 7 | 10.3 | 34 | 10.9 |
|  | Inpatient | 11 | 16.2 | 44 | 14.1 |
|  | Not currently practicing | 3 | 4.4 | 2 | 0.6 |
| What best describes your current practice environment? | |  |  |  |  |
|  | Private practice | 15 | 22.1 | 91 | 29.3 |
|  | Not-for profit system | 44 | 64.7 | 163 | 52.4 |
|  | For-profit system | 9 | 13.2 | 57 | 18.3 |
| Do you practice in an Academic Setting (overseeing learners)? | | |  |  |  |
|  | Yes | 32 | 47.8 | 165 | 52.7 |
|  | No | 35 | 52.2 | 148 | 47.3 |
