# Supplementary material for: Perceptions and attitudes toward artificial intelligence among frontline physicians and physicians’ assistants in Kansas: a cross-sectional survey
Source: JAMIA Open. 2024 Oct 7;7(4):ooae100. doi: 10.1093/jamiaopen/ooae100 (PMC11458514; doi:10.1093/jamiaopen/ooae100)
Supplement: ooae100_Supplementary_Data [file ooae100_supplementary_data.zip › Supplement 1 .docx]

**Appendix 1**. Comparison of responses by those who completed the survey versus those with incomplete surveys.

|  |  |  | **Survey response status** | | | |
| --- | --- | --- | --- | --- | --- | --- |
|  |  |  | **Complete** | | **Incomplete** | |
| **Question** | | **Response** | **n** | **%** | **n** | **%** |
| **Perceived Performance Anxiety** | |  |  |  |  |  |
|  | *I am concerned that the mechanisms used by AI-based devices may lead to inaccurate predictions* | |  |  |  |  |
|  |  | Strongly disagree | 11 | 2.8 | 3 | 2.4 |
|  |  | Disagree | 65 | 16.5 | 6 | 4.8 |
|  |  | Neutral | 75 | 19.0 | 25 | 20.0 |
|  |  | Agree | 181 | 45.9 | 34 | 27.2 |
|  |  | Strongly agree | 62 | 15.7 | 7 | 5.6 |
|  | *I am concerned that the mechanisms used by AI-based devices may result in medical errors* | |  |  |  |  |
|  |  | Strongly disagree | 10 | 2.5 | 3 | 2.4 |
|  |  | Disagree | 65 | 16.5 | 8 | 6.4 |
|  |  | Neutral | 61 | 15.5 | 17 | 13.6 |
|  |  | Agree | 187 | 47.5 | 38 | 30.4 |
|  |  | Strongly agree | 69 | 17.5 | 9 | 7.2 |
|  | *I am concerned that treatments provided by AI devices may be incomplete* | |  |  |  |  |
|  |  | Strongly disagree | 7 | 1.8 | 2 | 1.6 |
|  |  | Disagree | 53 | 13.5 | 4 | 3.2 |
|  |  | Neutral | 67 | 17.0 | 15 | 12.0 |
|  |  | Agree | 186 | 47.2 | 37 | 29.6 |
|  |  | Strongly agree | 79 | 20.1 | 16 | 12.8 |
|  | *I am concerned that the predictive models of AI-based tools may malfunction* | |  |  |  |  |
|  |  | Strongly disagree | 12 | 3.0 | 3 | 2.4 |
|  |  | Disagree | 67 | 17.0 | 6 | 4.8 |
|  |  | Neutral | 81 | 20.6 | 25 | 20.0 |
|  |  | Agree | 166 | 42.1 | 29 | 23.2 |
|  |  | Strongly agree | 63 | 16.0 | 11 | 8.8 |
|  | *I am concerned that the medical decisions made by AI devices may be inadequate* | |  |  |  |  |
|  |  | Strongly disagree | 7 | 1.8 | 1 | 0.8 |
|  |  | Disagree | 45 | 11.4 | 6 | 4.8 |
|  |  | Neutral | 58 | 14.7 | 9 | 7.2 |
|  |  | Agree | 194 | 49.2 | 42 | 33.6 |
|  |  | Strongly agree | 90 | 22.8 | 16 | 12.8 |
| **Perceived Social Biases** | |  |  |  |  |  |
|  | *I am concerned that the AI-based devices may overestimate or underestimate health risks in a certain patient population* | |  |  |  |  |
|  |  | Strongly disagree | 6 | 1.5 | 2 | 1.6 |
|  |  | Disagree | 41 | 10.4 | 7 | 5.6 |
|  |  | Neutral | 63 | 16.0 | 8 | 6.4 |
|  |  | Agree | 180 | 45.7 | 29 | 23.2 |
|  |  | Strongly agree | 100 | 25.4 | 10 | 8.0 |
|  | *I am concerned that data used in the AI devices may lead to societal discrimination to a certain patient group* | |  |  |  |  |
|  |  | Strongly disagree | 34 | 8.6 | 2 | 1.6 |
|  |  | Disagree | 99 | 25.1 | 15 | 12.0 |
|  |  | Neutral | 76 | 19.3 | 13 | 10.4 |
|  |  | Agree | 100 | 25.4 | 19 | 15.2 |
|  |  | Strongly agree | 83 | 21.1 | 8 | 6.4 |
|  | *I am concerned that AI-based tools used in healthcare may be unfair to a certain group of population* | |  |  |  |  |
|  |  | Strongly disagree | 35 | 8.9 | 3 | 2.4 |
|  |  | Disagree | 79 | 20.1 | 10 | 8.0 |
|  |  | Neutral | 76 | 19.3 | 12 | 9.6 |
|  |  | Agree | 121 | 30.7 | 20 | 16.0 |
|  |  | Strongly agree | 81 | 20.6 | 11 | 8.8 |
|  | *I am concerned that AI devices could lead to morally flawed practices in health care* | |  |  |  |  |
|  |  | Strongly disagree | 21 | 5.3 | 3 | 2.4 |
|  |  | Disagree | 78 | 19.8 | 10 | 8.0 |
|  |  | Neutral | 78 | 19.8 | 15 | 12.0 |
|  |  | Agree | 116 | 29.4 | 18 | 14.4 |
|  |  | Strongly agree | 97 | 24.6 | 12 | 9.6 |
|  | *Overall, I am concerned that the possibility of biases by AI devices to certain groups of the population is high* | |  |  |  |  |
|  |  | Strongly disagree | 28 | 7.1 | 4 | 3.2 |
|  |  | Disagree | 92 | 23.4 | 9 | 7.2 |
|  |  | Neutral | 93 | 23.6 | 18 | 14.4 |
|  |  | Agree | 95 | 24.1 | 18 | 14.4 |
|  |  | Strongly agree | 86 | 21.8 | 8 | 6.4 |
| **Perceived Privacy Concerns** | |  |  |  |  |  |
|  | *I think using AI-based applications helps health care entities collect too much personal information from me* | |  |  |  |  |
|  |  | Strongly disagree | 19 | 4.8 | 4 | 3.2 |
|  |  | Disagree | 124 | 31.5 | 13 | 10.4 |
|  |  | Neutral | 114 | 28.9 | 13 | 10.4 |
|  |  | Agree | 83 | 21.1 | 8 | 6.4 |
|  |  | Strongly agree | 52 | 13.2 | 3 | 2.4 |
|  |  |  |  |  |  |  |
|  | *I think in this case, I am concerned that health care entities use my health information for other purposes without my knowledge and authorization* | |  |  |  |  |
|  |  | Strongly disagree | 22 | 5.6 | 3 | 2.4 |
|  |  | Disagree | 104 | 26.4 | 11 | 8.8 |
|  |  | Neutral | 60 | 15.2 | 5 | 4.0 |
|  |  | Agree | 122 | 31.0 | 14 | 11.2 |
|  |  | Strongly agree | 84 | 21.3 | 8 | 6.4 |
|  | *In this case, I am concerned that my health information will be shared with other entities without my explicit consent* | |  |  |  |  |
|  |  | Strongly disagree | 23 | 5.8 | 3 | 2.4 |
|  |  | Disagree | 94 | 23.9 | 8 | 6.4 |
|  |  | Neutral | 70 | 17.8 | 7 | 5.6 |
|  |  | Agree | 119 | 30.2 | 15 | 12.0 |
|  |  | Strongly agree | 86 | 21.8 | 8 | 6.4 |
|  | *In this case, I am concerned that unauthorized people will have access to my health information* | |  |  |  |  |
|  |  | Strongly disagree | 28 | 7.1 | 3 | 2.4 |
|  |  | Disagree | 94 | 23.9 | 8 | 6.4 |
|  |  | Neutral | 65 | 16.5 | 7 | 5.6 |
|  |  | Agree | 120 | 30.5 | 14 | 11.2 |
|  |  | Strongly agree | 83 | 21.1 | 9 | 7.2 |
|  | *In this case, I am concerned about the privacy of my health information during AI-based health practices* | |  |  |  |  |
|  |  | Strongly disagree | 26 | 6.6 | 3 | 2.4 |
|  |  | Disagree | 98 | 24.9 | 10 | 8.0 |
|  |  | Neutral | 73 | 18.5 | 3 | 2.4 |
|  |  | Agree | 123 | 31.2 | 16 | 12.8 |
|  |  | Strongly agree | 68 | 17.3 | 7 | 5.6 |
|  | *In this case, I am concerned my health information would be sold to others without my permission* | |  |  |  |  |
|  |  | Strongly disagree | 27 | 6.9 | 3 | 2.4 |
|  |  | Disagree | 93 | 23.6 | 7 | 5.6 |
|  |  | Neutral | 61 | 15.5 | 6 | 4.8 |
|  |  | Agree | 118 | 29.9 | 16 | 12.8 |
|  |  | Strongly agree | 91 | 23.1 | 7 | 5.6 |
| **Perceived Trust in AI Mechanisms** | |  |  |  |  |  |
|  | *I trust in the AI-based clinical tools used for healthcare delivery* | |  |  |  |  |
|  |  | Strongly disagree | 40 | 10.2 | 2 | 1.6 |
|  |  | Disagree | 93 | 23.6 | 5 | 4.0 |
|  |  | Neutral | 160 | 40.6 | 18 | 14.4 |
|  |  | Agree | 95 | 24.1 | 5 | 4.0 |
|  |  | Strongly agree | 5 | 1.3 | 1 | 0.8 |
|  | *I trust in the AI algorithms used in the healthcare* | |  |  |  |  |
|  |  | Strongly disagree | 43 | 10.9 | 3 | 2.4 |
|  |  | Disagree | 84 | 21.3 | 3 | 2.4 |
|  |  | Neutral | 154 | 39.1 | 17 | 13.6 |
|  |  | Agree | 106 | 26.9 | 7 | 5.6 |
|  |  | Strongly agree | 4 | 1.0 | 1 | 0.8 |
|  | *I trust in AI s predictive and diagnostic ability for treatment purposes* |  |  |  |  |  |
|  |  | Strongly disagree | 46 | 11.7 | 2 | 1.6 |
|  |  | Disagree | 103 | 26.1 | 4 | 3.2 |
|  |  | Neutral | 126 | 32.0 | 19 | 15.2 |
|  |  | Agree | 105 | 26.6 | 4 | 3.2 |
|  |  | Strongly agree | 12 | 3.0 | 1 | 0.8 |
|  | *I trust in the accuracy and predictive powers of current AI algorithmic models used in the medical context* | |  |  |  |  |
|  |  | Strongly disagree | 50 | 12.7 | 2 | 1.6 |
|  |  | Disagree | 95 | 24.1 | 4 | 3.2 |
|  |  | Neutral | 145 | 36.8 | 19 | 15.2 |
|  |  | Agree | 93 | 23.6 | 5 | 4.0 |
|  |  | Strongly agree | 8 | 2.0 | 1 | 0.8 |
|  | *I trust that AI-based tools can adapt to specific and unforeseen medical situations.* | |  |  |  |  |
|  |  | Strongly disagree | 83 | 21.1 | 3 | 2.4 |
|  |  | Disagree | 117 | 29.7 | 4 | 3.2 |
|  |  | Neutral | 107 | 27.2 | 18 | 14.4 |
|  |  | Agree | 77 | 19.5 | 4 | 3.2 |
|  |  | Strongly agree | 8 | 2.0 | 2 | 1.6 |
| **Perceived Communication Barriers** | |  |  |  |  |  |
|  | *I am concerned that AI tools may eliminate the contact between healthcare professionals and patients* | |  |  |  |  |
|  |  | Strongly disagree | 16 | 4.1 | 1 | 0.8 |
|  |  | Disagree | 90 | 22.8 | 5 | 4.0 |
|  |  | Neutral | 42 | 10.7 | 4 | 3.2 |
|  |  | Agree | 130 | 33.0 | 9 | 7.2 |
|  |  | Strongly agree | 110 | 27.9 | 2 | 1.6 |
|  | *I am concerned that AI tools may reduce conversation between physicians and patients* | |  |  |  |  |
|  |  | Strongly disagree | 18 | 4.6 | 1 | 0.8 |
|  |  | Disagree | 78 | 19.8 | 5 | 4.0 |
|  |  | Neutral | 38 | 9.6 | 1 | 0.8 |
|  |  | Agree | 131 | 33.2 | 11 | 8.8 |
|  |  | Strongly agree | 127 | 32.2 | 3 | 2.4 |
|  | *I am concerned that AI devices may decrease human- aspects of relations in the medical contexts* | |  |  |  |  |
|  |  | Strongly disagree | 20 | 5.1 | 1 | 0.8 |
|  |  | Disagree | 60 | 15.2 | 4 | 3.2 |
|  |  | Neutral | 40 | 10.2 | 2 | 1.6 |
|  |  | Agree | 125 | 31.7 | 7 | 5.6 |
|  |  | **Strongly agree** | **145** | **36.8** | 7 | 5.6 |
|  | *I am concerned that by using AI devices, I may lose face-to-face cues and personal interactions with physicians* | |  |  |  |  |
|  |  | Strongly disagree | 21 | 5.3 | 1 | 0.8 |
|  |  | Disagree | 68 | 17.3 | 4 | 3.2 |
|  |  | Neutral | 34 | 8.6 | 3 | 2.4 |
|  |  | Agree | 125 | 31.7 | 7 | 5.6 |
|  |  | **Strongly agree** | **141** | **35.8** | 6 | 4.8 |
|  | *I am concerned that by using AI devices, I may be in a more passive position for making medical decisions* | |  |  |  |  |
|  |  | Strongly disagree | 17 | 4.3 | 1 | 0.8 |
|  |  | Disagree | 76 | 19.3 | 4 | 3.2 |
|  |  | Neutral | 53 | 13.5 | 3 | 2.4 |
|  |  | Agree | 136 | 34.5 | 12 | 9.6 |
|  |  | Strongly agree | 110 | 27.9 | 0 | 0.0 |
| **Perceived Unregulated Standard** | |  |  |  |  |  |
|  | *I am concern that special policies and guidelines for AI tools are not transparent yet* | |  |  |  |  |
|  |  | Strongly disagree | 2 | 0.5 | 0 | 0.0 |
|  |  | Disagree | 23 | 5.8 | 1 | 0.8 |
|  |  | Neutral | 67 | 17.0 | 3 | 2.4 |
|  |  | Agree | 169 | 42.9 | 7 | 5.6 |
|  |  | **Strongly agree** | **130** | **33.0** | 3 | 2.4 |
|  | *I am concerned that the safety and efficacy of AI medical tools are not regulated clearly* | |  |  |  |  |
|  |  | Strongly disagree | 3 | 0.8 | 0 | 0.0 |
|  |  | Disagree | 30 | 7.6 | 1 | 0.8 |
|  |  | Neutral | 63 | 16.0 | 4 | 3.2 |
|  |  | Agree | 166 | 42.1 | 7 | 5.6 |
|  |  | **Strongly agree** | **131** | **33.2** | 2 | 1.6 |
|  | *I am concerned that regulatory standards to assess AI algorithmic safety are yet to be formalized* | |  |  |  |  |
|  |  | Strongly disagree | 3 | 0.8 | 0 | 0.0 |
|  |  | Disagree | 21 | 5.3 | 0 | 0.0 |
|  |  | Neutral | 56 | 14.2 | 6 | 4.8 |
|  |  | Agree | 187 | 47.5 | 6 | 4.8 |
|  |  | Strongly agree | 123 | 31.2 | 2 | 1.6 |
|  | *I am concerned that appropriate regulatory and accreditation system regarding AI-based devices is not in place yet* | |  |  |  |  |
|  |  | Strongly disagree | 6 | 1.5 | 0 | 0.0 |
|  |  | Disagree | 23 | 5.8 | 0 | 0.0 |
|  |  | Neutral | 53 | 13.5 | 6 | 4.8 |
|  |  | Agree | 180 | 45.7 | 6 | 4.8 |
|  |  | Strongly agree | 128 | 32.5 | 2 | 1.6 |
|  | *I am concerned about the lack of clear guidelines to monitor the performance of AI tools in the medical context* | |  |  |  |  |
|  |  | Strongly disagree | 5 | 1.3 | 0 | 0.0 |
|  |  | Disagree | 27 | 6.9 | 0 | 0.0 |
|  |  | Neutral | 53 | 13.5 | 6 | 4.8 |
|  |  | Agree | 170 | 43.1 | 5 | 4.0 |
|  |  | **Strongly agree** | **135** | **34.3** | 2 | 1.6 |
| **Perceived Liability Issues** | |  |  |  |  |  |
|  | *I am concerned because it is not clear who is responsible when errors result from the use of AI clinical tools* | |  |  |  |  |
|  |  | Strongly disagree | 4 | 1.0 | 0 | 0.0 |
|  |  | Disagree | 37 | 9.4 | 0 | 0.0 |
|  |  | Neutral | 52 | 13.2 | 1 | 0.8 |
|  |  | Agree | 157 | 39.8 | 3 | 2.4 |
|  |  | **Strongly agree** | **142** | **36.0** | 2 | 1.6 |
|  | *I am concerned about the liability of using AI-based services for my healthcare* | |  |  |  |  |
|  |  | Strongly disagree | 5 | 1.3 | 0 | 0.0 |
|  |  | Disagree | 43 | 10.9 | 0 | 0.0 |
|  |  | Neutral | 60 | 15.2 | 1 | 0.8 |
|  |  | Agree | 150 | 38.1 | 3 | 2.4 |
|  |  | **Strongly agree** | **135** | **34.3** | 3 | 2.4 |
|  | *I am concerned because it is not clear who becomes responsible if AI-based tools offer wrong recommendations* | |  |  |  |  |
|  |  | Strongly disagree | 5 | 1.3 | 0 | 0.0 |
|  |  | Disagree | 41 | 10.4 | 0 | 0.0 |
|  |  | Neutral | 54 | 13.7 | 1 | 0.8 |
|  |  | Agree | 149 | 37.8 | 2 | 1.6 |
|  |  | **Strongly agree** | **142** | **36.0** | 4 | 3.2 |
|  | *I am concerned because it is unclear where the lines of responsibility begin or end when AI devices guide clinical care* | |  |  |  |  |
|  |  | Strongly disagree | 7 | 1.8 | 0 | 0.0 |
|  |  | Disagree | 34 | 8.6 | 0 | 0.0 |
|  |  | Neutral | 54 | 13.7 | 1 | 0.8 |
|  |  | Agree | 164 | 41.6 | 3 | 2.4 |
|  |  | **Strongly agree** | **130** | **33.0** | 3 | 2.4 |
|  | *I am concerned because it is not clear who is responsible if appropriate AI-recommended treatment options are mistakenly dismissed* | |  |  |  |  |
|  |  | Strongly disagree | 7 | 1.8 | 0 | 0.0 |
|  |  | Disagree | 38 | 9.6 | 0 | 0.0 |
|  |  | Neutral | 60 | 15.2 | 1 | 0.8 |
|  |  | Agree | 160 | 40.6 | 3 | 2.4 |
|  |  | Strongly agree | 125 | 31.7 | 3 | 2.4 |
|  | *Overall, I am concerned that the use of AI clinical tools for clinical purposes increases my liability* | |  |  |  |  |
|  |  | Strongly disagree | 9 | 2.3 | 0 | 0.0 |
|  |  | Disagree | 59 | 15.0 | 0 | 0.0 |
|  |  | Neutral | 75 | 19.0 | 2 | 1.6 |
|  |  | Agree | 131 | 33.2 | 2 | 1.6 |
|  |  | Strongly agree | 116 | 29.4 | 3 | 2.4 |
| **Perceived Risks (very low/very high)** | |  |  |  |  |  |
|  | *The risk of using AI-based tools for medical purposes is* | |  |  |  |  |
|  |  | Very low | 10 | 2.5 | 0 | 0.0 |
|  |  | Low | 88 | 22.3 | 1 | 0.8 |
|  |  | Moderate | 157 | 39.8 | 1 | 0.8 |
|  |  | High | 90 | 22.8 | 0 | 0.0 |
|  |  | Very high | 42 | 10.7 | 0 | 0.0 |
|  | *The degree of uncertainty associated with the use of AI clinical tools is* | |  |  |  |  |
|  |  | Very low | 7 | 1.8 | 0 | 0.0 |
|  |  | Low | 57 | 14.5 | 1 | 0.8 |
|  |  | Moderate | 153 | 38.8 | 0 | 0.0 |
|  |  | High | 99 | 25.1 | 1 | 0.8 |
|  |  | Very high | 72 | 18.3 | 0 | 0.0 |
|  | *The potential loss associated with the use of AI devices is* | |  |  |  |  |
|  |  | Very low | 16 | 4.1 | 0 | 0.0 |
|  |  | Low | 103 | 26.1 | 1 | 0.8 |
|  |  | Moderate | 136 | 34.5 | 1 | 0.8 |
|  |  | High | 80 | 20.3 | 0 | 0.0 |
|  |  | Very high | 53 | 13.5 | 0 | 0.0 |
|  | *The likelihood of unexpected problems with the use of AI devices is* | |  |  |  |  |
|  |  | Very low | 4 | 1.0 | 0 | 0.0 |
|  |  | Low | 48 | 12.2 | 1 | 0.8 |
|  |  | Moderate | 129 | 32.7 | 0 | 0.0 |
|  |  | High | 117 | 29.7 | 1 | 0.8 |
|  |  | Very high | 89 | 22.6 | 0 | 0.0 |
|  | *Overall, the chance of adverse consequences associated with the use of AI-based tools for healthcare purposes is* | |  |  |  |  |
|  |  | Very low | 9 | 2.3 | 0 | 0.0 |
|  |  | Low | 86 | 21.8 | 1 | 0.8 |
|  |  | Moderate | 147 | 37.3 | 1 | 0.8 |
|  |  | High | 84 | 21.3 | 0 | 0.0 |
|  |  | Very high | 62 | 15.7 | 0 | 0.0 |
| **Perceived Benefits** | |  |  |  |  |  |
|  | *I believe AI-based services can improve diagnostics* | |  |  |  |  |
|  |  | Strongly disagree | 13 | 3.3 | 0 | 0.0 |
|  |  | Disagree | 22 | 5.6 | 0 | 0.0 |
|  |  | Neutral | 64 | 16.2 | 0 | 0.0 |
|  |  | Agree | 219 | 55.6 | 1 | 0.8 |
|  |  | Strongly agree | 75 | 19.0 | 0 | 0.0 |
|  | *I think AI-based devices can enhance prognosis* | |  |  |  |  |
|  |  | Strongly disagree | 18 | 4.6 | 0 | 0.0 |
|  |  | Disagree | 33 | 8.4 | 0 | 0.0 |
|  |  | Neutral | 123 | 31.2 | 0 | 0.0 |
|  |  | Agree | 167 | 42.4 | 1 | 0.8 |
|  |  | Strongly agree | 49 | 12.4 | 0 | 0.0 |
|  | *I believe AI-based devices can advance patient management systems* | |  |  |  |  |
|  |  | Strongly disagree | 11 | 2.8 | 0 | 0.0 |
|  |  | Disagree | 24 | 6.1 | 0 | 0.0 |
|  |  | Neutral | 104 | 26.4 | 0 | 0.0 |
|  |  | Agree | 192 | 48.7 | 1 | 0.8 |
|  |  | Strongly agree | 59 | 15.0 | 0 | 0.0 |
|  | *I believe AI-based tools can suggest accurate care planning* | |  |  |  |  |
|  |  | Strongly disagree | 12 | 3.0 | 0 | 0.0 |
|  |  | Disagree | 35 | 8.9 | 0 | 0.0 |
|  |  | Neutral | 116 | 29.4 | 0 | 0.0 |
|  |  | Agree | 178 | 45.2 | 1 | 0.8 |
|  |  | Strongly agree | 46 | 11.7 | 0 | 0.0 |
|  | *I think AI-based services can recommend reliable treatment options* | |  |  |  |  |
|  |  | Strongly disagree | 13 | 3.3 | 0 | 0.0 |
|  |  | Disagree | 32 | 8.1 | 0 | 0.0 |
|  |  | Neutral | 117 | 29.7 | 0 | 0.0 |
|  |  | Agree | 189 | 48.0 | 1 | 0.8 |
|  |  | Strongly agree | 42 | 10.7 | 0 | 0.0 |
|  | *I think AI-based tools can reduce healthcare costs* | |  |  |  |  |
|  |  | Strongly disagree | 48 | 12.2 | 0 | 0.0 |
|  |  | Disagree | 71 | 18.0 | 0 | 0.0 |
|  |  | Neutral | 115 | 29.2 | 0 | 0.0 |
|  |  | Agree | 115 | 29.2 | 1 | 0.8 |
|  |  | Strongly agree | 43 | 10.9 | 0 | 0.0 |
|  | *Overall, I think AI-based devices can boost healthcare outcomes* | |  |  |  |  |
|  |  | Strongly disagree | 31 | 7.9 | 0 | 0.0 |
|  |  | Disagree | 46 | 11.7 | 0 | 0.0 |
|  |  | Neutral | 133 | 33.8 | 0 | 0.0 |
|  |  | Agree | 137 | 34.8 | 1 | 0.8 |
|  |  | Strongly agree | 45 | 11.4 | 0 | 0.0 |
| **Intention to Use AI-based Tools** | |  |  |  |  |  |
|  | *I agree to use AI-based tools for clinical purposes* | |  |  |  |  |
|  |  | Strongly disagree | 42 | 10.7 | 0 | 0.0 |
|  |  | Disagree | 58 | 14.7 | 0 | 0.0 |
|  |  | Neutral | 138 | 35.0 | 0 | 0.0 |
|  |  | Agree | 124 | 31.5 | 1 | 0.8 |
|  |  | Strongly agree | 30 | 7.6 | 0 | 0.0 |
|  | *Using AI-based tools for healthcare purposes is something I would consider* | |  |  |  |  |
|  |  | Strongly disagree | 23 | 5.8 | 0 | 0.0 |
|  |  | Disagree | 29 | 7.4 | 0 | 0.0 |
|  |  | Neutral | 80 | 20.3 | 0 | 0.0 |
|  |  | Agree | 200 | 50.8 | 1 | 0.8 |
|  |  | Strongly agree | 59 | 15.0 | 0 | 0.0 |
|  | *I would like to use AI-based devices to manage my healthcare* | |  |  |  |  |
|  |  | Strongly disagree | 53 | 13.5 | 0 | 0.0 |
|  |  | Disagree | 78 | 19.8 | 0 | 0.0 |
|  |  | Neutral | 120 | 30.5 | 0 | 0.0 |
|  |  | Agree | 106 | 26.9 | 1 | 0.8 |
|  |  | Strongly agree | 36 | 9.1 | 0 | 0.0 |
|  | *In the future, I am willing to use AI-based services for diagnostics and treatments* | |  |  |  |  |
|  |  | Strongly disagree | 34 | 8.6 | 0 | 0.0 |
|  |  | Disagree | 40 | 10.2 | 0 | 0.0 |
|  |  | Neutral | 131 | 33.2 | 0 | 0.0 |
|  |  | Agree | 145 | 36.8 | 1 | 0.8 |
|  |  | Strongly agree | 40 | 10.2 | 0 | 0.0 |
|  | *I am very likely to use recommendations provided by AI-based tools for care planning* | |  |  |  |  |
|  |  | Strongly disagree | 39 | 9.9 | 0 | 0.0 |
|  |  | Disagree | 63 | 16.0 | 0 | 0.0 |
|  |  | Neutral | 166 | 42.1 | 0 | 0.0 |
|  |  | Agree | 90 | 22.8 | 1 | 0.8 |
|  |  | Strongly agree | 32 | 8.1 | 0 | 0.0 |
